# Expanding the role of bitter taste receptor in extra oral tissues: TAS2R38 is expressed in human adipocytes

**DOI:** 10.1080/21623945.2019.1709253

**Published:** 2020-01-03

**Authors:** Raffaella Cancello, Giancarlo Micheletto, Dorela Meta, Rosalia Lavagno, Emanuele Bevilacqua, Valerio Panizzo, Cecilia Invitti

**Affiliations:** a Laboratorio di ricerche sull’Obesità, Istituto Auxologico Italiano, IRCCS, Milano, Italy; b Dipartimento di Chirurgia Generale, Istituto Clinico Sant’Ambrogio, Milano, Italy; c Dipartimento di Fisiopatologia medico-chirurgica e dei trapianti (DEPT), Università degli Studi di Milano, Milan, Italy

**Keywords:** Adipocytes, adipose tissue, lipolysis, obesity, TAS2R38

## Abstract

Increasing evidence indicates that taste receptors mediate a variety of functions in extra-oral tissues. The present study investigated the expression of bitter taste receptor TAS2R38 in human adipocytes, the possible link with genetic background and the role of TAS2R38 in cell delipidation and lipid accumulation rate *in vitro*. Subcutaneous (SAT) and visceral (VAT) adipose tissues were collected in 32 obese and 18 lean subjects. The TAS2R38 gene expression and protein content were examined in whole tissues, differentiated adipocytes and stroma-vascular fraction cells (SVF). The P49A SNP of TAS2R38 gene was determined in each collected sample. The effect of two bitter agonists (6-n-propylthiouracil and quinine) was tested. TAS2R38 mRNA was more expressed in SAT and VAT of obese than lean subjects and the expression/protein content was greater in mature adipocytes. The expression levels were not linked to P49A variants. In *in vitro* differentiated adipocytes, bitter agonists induced a significant delipidation. Incubation with 6-n-propylthiouracil induced an inhibition of lipid accumulation rate together with an increase in TAS2R38 and a decrease in genes involved in adipocyte differentiation. In conclusion, TAS2R38 is more expressed in adipocytes of obese than lean subjects and is involved in differentiation and delipidation processes.

## Introduction

The sense of taste acts as a nutrient-sensing system and may be considered a kind of detection system, informing us of suitability to swallow or reject food. Any alteration of this system might thus contribute to an excessive energy intake and weight gain [1]. Taste information are perceived in the gustatory papillae of the oral cavity by specific taste receptors for bitter, sweet, and umami, that belong to the superfamily of G-protein-coupled receptors, and salt and sour receptors, which are ion-sensing channels [2]. Some evidence demonstrate that overweight/obese subjects have a lower sensitivity to all taste stimuli than normal weight individuals [3,4] suggesting that the higher taste threshold may be involved in the excessive consumption of food. This finding, however, has not been shared by other studies due to the different techniques used and the confounding factors affecting taste perception (i.e. sex, age, smoking/drinking habits, experiences, socio-cultural habits and genetic differences). Thus, it has not been established yet whether the alterations of taste sensitivity are the cause of overeating or represent the consequence of incorrect eating habits established with the progression of obesity [5,6]. The most common way to evaluate the individual variation in taste is to assess the ability to taste the bitter compound 6-n-propylthiouracil (PROP). PROP sensitivity is considered a proxy of general taste ability as it is associated with the sensitivity to other oro-sensory stimuli [5].

The ability to perceive the bitterness of PROP is a heritable trait and the gene associated with bitter PROP perception is TAS2R38 [7]. Three common allelic isoforms of TAS2R38 gene identify super-taster (PAV/PAV), taster (PAV/AVI) and non-taster subjects (AVI/AVI) and the first variant site (P49A) explain large part of the phenotypic variation in PROP perception [7–10]. However, several data demonstrated that TAS2R38 gene polymorphisms only partially explain the variance in bitter perception to which other non-genetic factors may contribute [11,12].

Over the past decade, it has become evident that taste receptors are expressed not only in taste buds of the tongue surface but also in extra gustatory organs (such as: digestive, respiratory, genitourinary systems, heart, brain, thyroid, skin, placenta and immune cells) suggesting that different cell type, outside the oral cavity, may use taste receptors [13–15]. The exact role of TAS2R38 outside the gustatory systems is still elusive.

The evidence that obese individuals express more TAS2R38 immunoreactive cells in colonic mucosa than lean subjects [16] and that in mice the intragastric administration of bitter chemicals alters food intake and body weight through ghrelin release [17], support the hypothesis that taste receptors could be involved in the regulation of appetite hormones secretion, energy balance and body weight regulation. Sweet and bitter taste receptors are expressed in mice adipocytes and affect adipogenesis, even if in a direction not yet clear [18–21]. No data are available on the presence and hypothetical function of TAS2R38 in human adipose tissue/adipocytes.

We here investigated the expression of TAS2R38 in human subcutaneous (SAT) and visceral adipose tissue (VAT) from obese and normal weight individuals and its relation with the P49A variants. In addition, we assessed the *in vitro* effect of two different bitter agonists on lipid metabolism.

## Materials and methods

### Adipose tissue samples

The Ethics Committee of the IRCCS Istituto Auxologico Italiano (Milan, Italy) approved the study (https://www.auxologico.it/ricerca-formazione/comitato-etico, approval CE code: 2017_05_16_08) and all subjects gave their written informed consent after a full explanation of the study. We collected biopsies of subcutaneous (SAT) and visceral (VAT) adipose tissue from a total of 50 non-diabetic subjects: 32 obese subjects (20 women, 12 men, age 45.1 ± 10.9 years, BMI 43.1 ± 9.2 kg/m^2^) who underwent bariatric surgery procedures (such as sleeve gastrectomy, intestinal by pass, gastric banding) and 18 normal weight individuals (11 women and 7 men, age 43.5 ± 14.1 years, BMI 24.2 ± 2.3 Kg/m^2^) free from inflammatory, infective or neoplastic diseases submitted to aesthetic plastic surgery procedures.

### DNA and RNA extraction and cDNA synthesis

From each collected biopsy, we isolated DNA and RNA. The biopsies were homogenized by a mechanical disruption step using the IKA T10 Ultra Turrax (IKA) with a lysis step using ultra-high-density Bashing Beads according to the manufacturer’s instructions (Zymo research), then total DNA was then extracted with DNA Blood & Tissue Kit following manufacturer instructions (Qiagen). The RNA was extracted with RNeasy mini kit following manufacturer instructions (Qiagen). The RNase-Free DNase Set (Qiagen) was used for digestion of possible residual DNA during RNA purification using RNeasy mini Kits in order to guarantee a complete DNA removal from RNA samples. Amounts and quality of the extracted DNA/RNA was assessed by NanoDropH ND-1000 spectrophotometer (NanoDrop Technologies). The cDNA was obtained by reverse-transcription of 500 ng extracted RNA, using the SuperScript VILO cDNA Synthesis Kit and Master Mix (Life Technologies).

### Real-time quantitative PCR (RTqPCR)

TAS2R38, fatty acid synthase (FASN), peroxisome proliferator-activated receptor gamma (PPARγ) and glucose transporter 4 (GLUT4) gene expression levels were assessed starting from 10 ng of cDNA by using TaqMan probes (assay on demand, Applied Biosystems). The housekeeping gene RPLP0 (human ribosomal protein LP0) was used for data normalization due to the high stability of expression. Data were analysed with the SDS V.3 software (Software Diversified Systems) and the relative quantification, expressed as arbitrary units (AU), was calculated using the 2^-ΔΔCt method.

### Protein extraction and western blotting

Proteins were extracted in isolated mature adipocytes (A), in stroma vascular fraction (SVF) cells and in 10 days *in vitro* differentiated adipocytes (DA). Cell and/or tissue fragments were homogenized in ice-cold RIPA buffer with freshly added protease inhibitor cocktail (Roche), then incubated on ice for 30 min and centrifuged at 12,000 x g for 30 min at 4°C. The supernatants were collected, and total protein concentrations were quantified using Bovine Serum Albumin standard curve (Thermo Scientific). A total of 20 µg of extracted proteins were separated and transferred to a nitrocellulose membrane as previously described [22]. After blocking with 10% bovine serum albumin (BSA, Sigma) for 1 h, membranes were incubated overnight in primary antibodies at 4°C. The primary antibody was rabbit polyclonal anti-human TAS2R38 (Thermo Fisher scientific, diluted 1:2,000 with 5% BSA) tested for western blotting applications and mouse monoclonal β-actin antibody (Fine Test Biotech Co, Ltd., diluted 1:10,000 with 5% BSA) was used for protein loading control. After incubation, membranes were exposed to 1:10,000 HRP-conjugated goat anti-rabbit IgG (H + L) or for β-actin 1:5,000 goat anti-mouse IgM secondary antibodies for 1 h at RT. The signals were quantified by densitometric procedures and expressed as arbitrary units (AU) after normalization on β-actin content. The membranes were treated for chemiluminescence detection (Novex ECL, HRP Chemiluminescent Substrate Reagent Kit, Invitrogen) and after exposure to X-ray films (Amersham Hyperfilm ECL), the signal obtained was acquired and analysed by the ImageJ software [23].

### TAS2R38 genotyping

The P49A genotype of TAS2R38 gene was analysed in all samples by restriction fragment length polymorphisms (RFLP) method, as previously described [24]. A total of 500 ng of extracted DNA was amplified with forward (5ʹ-CCT TCG TTT CTT GGT GAA TTT TTG GGA TGT AGT GAA GAG GCGG-3ʹ) and reverse (5ʹ-AGG TTG GCT TGG TTT GCA ATC ATC-3ʹ) primers for human TAS2R38 by PCR thermal Cycler (Applied Biosystems). The amplification step was programmed for 30 cycles as follows: 94°C for 30 s (Denaturing Step), 64°C for 45 s (Annealing step) and 72°C for 45 s (Extending Step) before finishing with an extension step of 72°C for 5 min. Ten microlitres of amplified fragment (221 bp) was digested with restriction enzyme HaeIII for 2 h at 37°C, then incubated at 80° for 20ʹ and analysed by gel electrophoresis (4% agarose) with 1X TAE Buffer. Size markers (100 bp DNA ladder, BioLabs) were loaded into the far left lane of the gel to check the fragment sizes. The gel was stained by adding 1 μL of ethidium bromide and ran at 120 V for 45 min and results were recorded using a transilluminator photograph (Biodoc TM). The tt homozygote yielded the 221 bp uncut fragment only (considered as non-taster genotype), the Tt heterozygote yielded three bands (221, 177 and 44 bp) (considered as medium taster genotype), while TT homozygote yielded two bands of 177 and 44 bp (considered as super-taster genotype).

### In vitro cell cultures

Fragments of collected SAT/VAT samples were digested with 1 mg/ml collagenase type II (Sigma) as previously described [25]. Stromal vascular fraction cells were isolated by centrifugation and cultured at 37°C in a 5% CO_2_ incubator with 1:1 Ham’s F12/DMEM (Invitrogen) supplemented with 10% decomplemented Foetal Bovine Serum (FBS) (Sigma), penicillin, streptomycin and amphotericin B (Invitrogen). Isolated SVF cells were counted with an automated Beckmann cell counter Z2 (Beckman) after dilution in Isoton 4 buffer (Beckman) and then seeded. When the SVF cells reached the confluence the differentiation was started and continued for 10 days with Stem Pro medium (Invitrogen) without adding FBS. The culture of the primary adipose SVF cells was performed following standard protocols [26]. To assess delipidation, 10 days differentiated cells (containing intracellular triglycerides) were starved and then stimulated for 4 h (acute stimulus) with three different concentrations of bitter receptor agonists: 20, 50, and 100 μM of Quinine (Sigma) and 1, 10, and 20 μM of 6-n-propylthiouracil (PROP) (Sigma). Four hours Caffeine (Sigma, 20, 50, and 100 μM) stimulus was used as delipidation control, due to its known strong lipolytic effect through inhibition of phosphodiesterase, stimulation of β-adrenergic receptors and increase in cyclic adenosine monophosphate (AMP) [27]. Quinine, caffeine and PROP were dissolved in distilled sterile water. The sterile water volume used as dilution vehicle was added in the respective untreated controls. For the lipid accumulation rate (= *in vitro* adipose differentiation process), SVF cells at confluence were differentiated in Stem Pro medium (Invitrogen) with or without adding 10 µM PROP for a 10-day chronic stimulus. In unstimulated cells (controls) the volume of sterile water used to dilute PROP was added. Every 2 days, cells were collected for the assessment of the intracellular lipid content and the RNA extracted for the TAS2R38, FASN, PPARγ and GLUT4 gene expression detection by RTqPCR, as previously described. The intracellular lipid content was assessed by Victor 3TM 1420 Multilabel fluorescence counter (Perkin Elmer) using AdipoRed assay reagent (Lonza) and following manufacturer instructions. Data were expressed as arbitrary fluorescence units (AFU). Cell viability was assessed by Trypan Blue Solution 0.4% staining (Gibco, Invitrogen) and inverted microscope (Carl Zeiss) observation.

### Statistical analysis

The statistical analysis was carried out using SPSS software (IBM Corp. Released 2017. IBM SPSS Statistics for Windows, Version 25.0. Armonk, NY: IBM Corp) and GraphPad Prism. Data are expressed as mean±standard error (SE). Means were compared by one-way ANOVA. The frequencies of P49A gene variants of TAS2R38 were compared using the χ-square test. Group multiple comparisons were made using the one-way ANOVA, two-way ANOVA or two-way Repeated Measure (RM) ANOVA followed by Bonferroni post hoc test, as appropriate. A p-value of <0.05 was considered statistically significant.

## Results

### Gene expression and protein levels of TAS2R38 in adipose tissue

**Figure 1.**
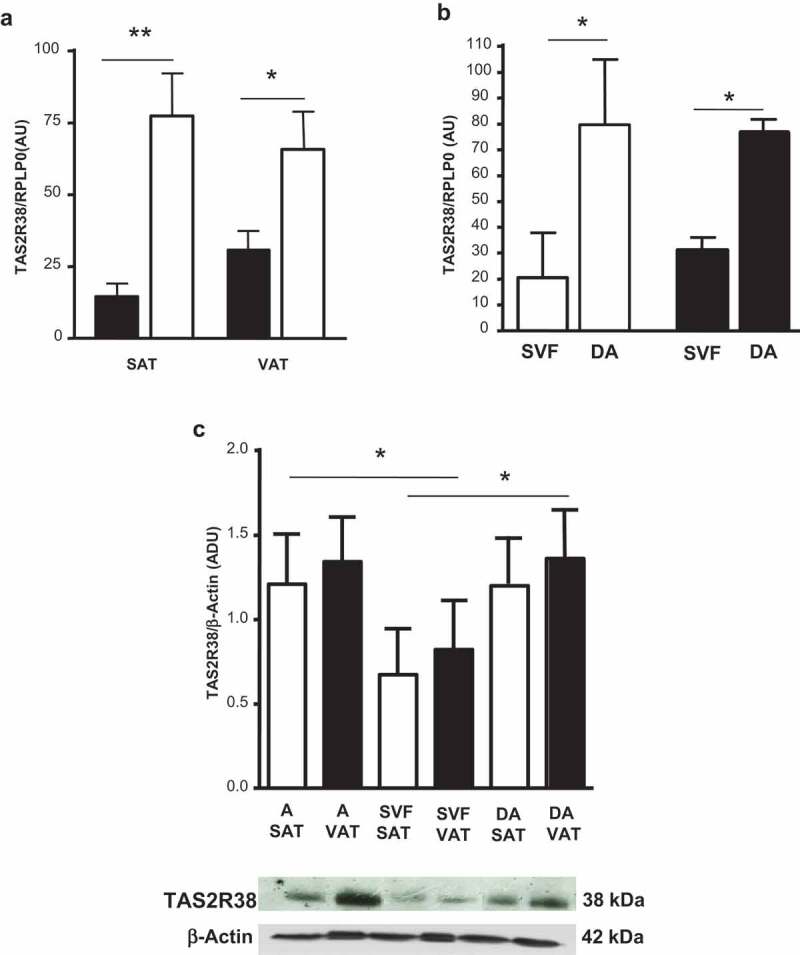
TAS2R38 mRNA gene expression in whole subcutaneous (SAT) and visceral (VAT) adipose tissue of lean subjects (black bars) versus obese patients (white bars) (panel a, OB-SAT *vs* NW-SAT, **p < 0.01 and OB-VAT *vs* NW-VAT, *p < 0.05). TAS2R38 gene expression in *in vitro* differentiated adipocytes (DA) and stroma-vascular fraction cells (SVF) from SAT (white bars, SVF *vs* DA, *p < 0.05) and VAT (black bars, SVF *vs* DA, *p < 0.05) of obese patients (panel b). The gene expression is reported as arbitrary units (AU) and normalized by gene expression of RPLP0 (housekeeping gene) (panels a–b). TAS2R38 protein quantification in isolated adipocytes (A), SVF cells and in vitro DA from SAT (white bars) and VAT (black bars) of obese patients by western blotting and densitometric analysis (Arbitrary Densitometric Units, ADU, A, DA *vs* SVF, *p < 0.05) (c, upper panel). The TAS2R38 and β-Actin protein immuno-detection in one representative blot is shown (c, lower panel)

The TAS2R38 mRNA was more expressed in whole SAT and VAT of obese than lean subjects (p < 0.05) (Figure 1(a)), and was not significantly different between sexes in both adipose tissue depots (not shown). In obese subjects, TAS2R38 gene expression was greater in the *in vitro* differentiated adipocytes than in isolated SVF cells (Figure 1(b)). Protein levels of TAS2R38 in isolated and in *in vitro* differentiated adipocytes were 1.6 and 1.7 higher than in SVF cells in both adipose tissue depots (Figure 1(c)).

### Genotype variants and TAS2R38 expression

**Figure 2.**
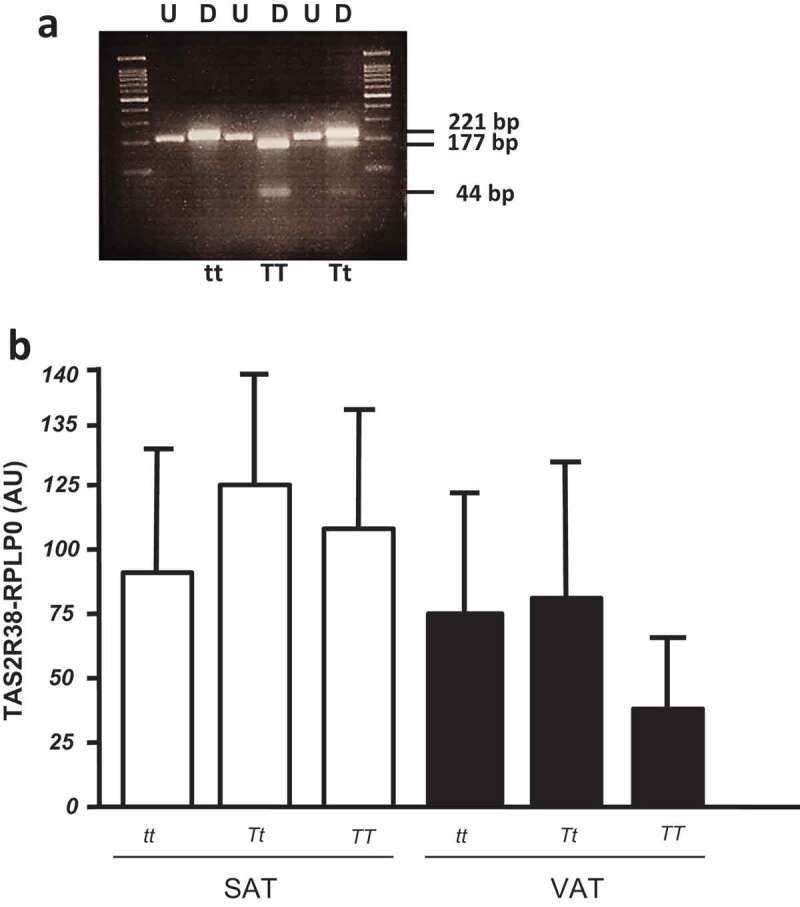
(a) Restriction fragment length polymorphism (RFLP) analysis of TAS gene for P49A SNP. The undigested (U) and *HaeIII* digested (D) bands are shown in a 4% agarose gel. The DNA fragments of 221 bp, 177 bp, and 44 bp are indicated. In the first and last gel lines, the 100 bp-DNA ladder was loaded. (b) TAS2R38 gene expression by RTqPCR in obese SAT and VAT biopsies by P49A-TAS2R38 gene variants: super taster (*TT*), taster (*Tt*) and non-taster (*tt*). The gene expression is reported as arbitrary units (AU) after normalization with RPLP0 expression (multiple comparisons, *NS*)

The P49A variants of TAS2R38 gene were analysed in a total of 47 adipose tissue samples by RFLP (the analysis was not suitable in three samples, due to low DNA quality) (Figure 2 (a)). The frequencies of TT, Tt, and tt were, respectively, 24%, 48%, and 28%. Taking into account the small sample size of the considered cohort, there was no significant difference in the P49A genotypes between normal weight and obese patients, nor in males/females subjects. The TAS2R38 gene expression was not different among the three P49A gene variants, both in SAT and VAT depots of all studied subjects (Figure 2(b)), as well as in the subset of SAT and VAT samples derived from obese patients (*not shown*).

### Effect of bitter taste agonists on adipocyte biology

**Figure 3.**
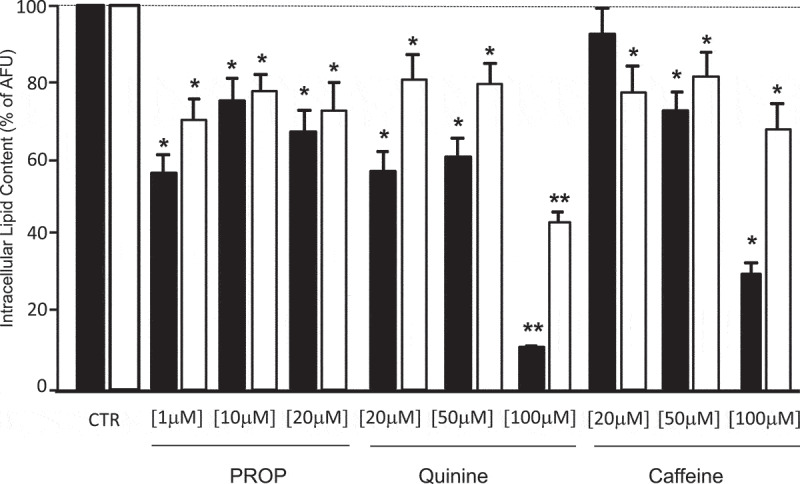
Delipidation (expressed as % of arbitrary fluorescence units, AFU) in *in vitro* differentiated adipocytes from subcutaneous (SAT, black bars) and visceral (VAT, white bars) adipose tissue after stimulation with three different concentrations of PROP, quinine and caffeine (used as delipidation control). * p < 0.05 and **p < 0.01 *vs* untreated control (CTR)

**Figure 4.**
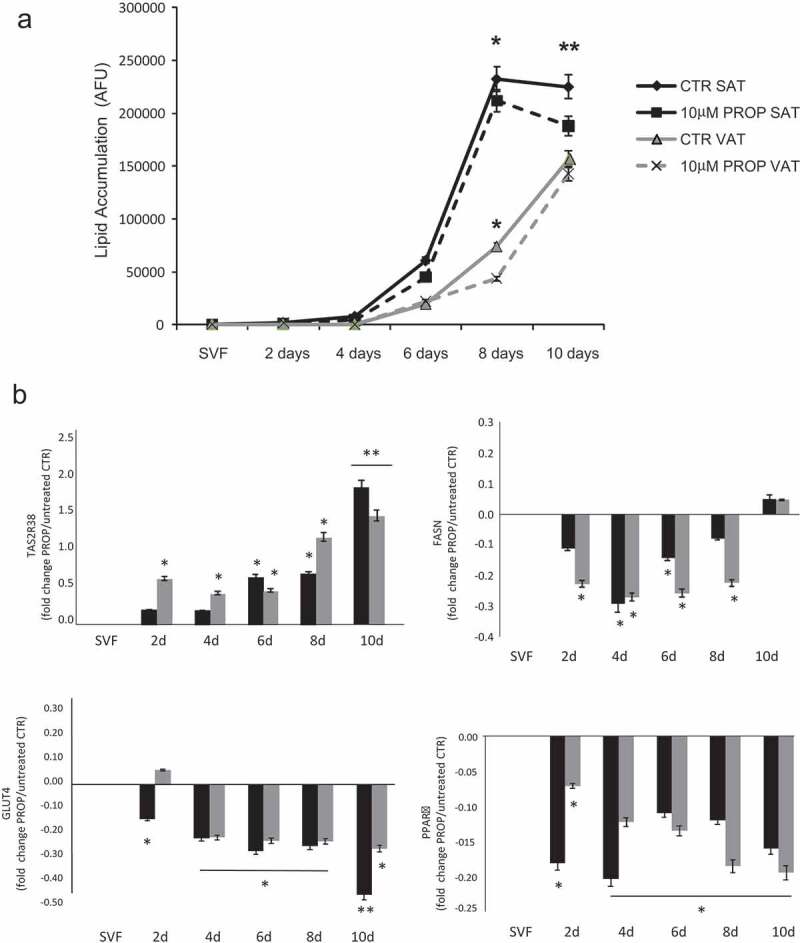
Time course of lipid accumulation rate (arbitrary fluorescence units, AFU) observed in *in vitro* SAT (black lines) and VAT (grey lines) derived stroma-vascular cells (SVF) during 10 days differentiation into mature adipocytes with 10 µM PROP (dotted lines) and without stimulation (differentiation control, CTR, continuous lines) (panel a). * p < 0.05 and **p < 0.01 *vs* CTR. Gene expression of TAS2R38, FASN GLUT4 and PPARγ during 10 days in vitro differentiation into mature adipocytes in SAT derived (black blocks) and VAT derived (grey blocks) cells with/without 10 µM PROP stimulation (panel b). The gene expression is reported as fold change (mean±SE) of stimulated/unstimulated cells (* p < 0.05, **p < 0.01, 10 µM PROP treated cells *v*s untreated control (SVF) of the correspondent time point at 2, 4, 6, 8 and 10 days (d) of differentiation)

The *in vitro* differentiated adipocytes were stimulated with three different concentrations of PROP, quinine and caffeine (used as delipidation control) for 4 h (acute stimulus). All compounds induced a significant intracellular delipidation compared to the respective untreated cells, with a greater effect in SAT-derived adipocytes (p < 0.05). Differently to caffeine in SAT adipocytes (p < 0.05, 20 *vs* 50 and 100 µM stimulation) there were no significant differences between the lipolytic effect of the three concentrations of PROP and quinine used (Figure 3). At the highest quinine concentration used (100 µM), there was the greater delipidation rate which was however associated with a significant cytotoxic effect (cell death in 30% of quinine stimulated adipocytes) (Figure 3). The chronic incubation of SVF cells with 10 µM PROP during the 10 days differentiation decreased the lipid accumulation from the sixth day onwards reaching the lowest level at day 10 in SAT-derived adipocytes (Figure 4(a)). The 10 µM PROP-induced decrease in lipid accumulation was associated with the significant increase in TAS2R38 expression (p < 0.05) and the decrease in FASN, PPARγ, GLUT4 expression in SAT and VAT derived cells under differentiation (p < 0.05, Figure 4(b)).

## Discussion

We demonstrated for the first time that TAS2R38 is present both at RNA and protein level in human adipocytes with highest expression levels in the adipose tissue of obese than lean individuals. The TAS2R38 expression in SAT and VAT is similar among subjects with the three P49A genotype variants, suggesting that the expression of this bitter receptor in human adipocytes is not influenced by this genetic component. This result agrees with what observed in sino-nasal tissues and tongue papillae where genotype variants of TAS2R38 did not affect the mRNA expression of the receptor [14]. Furthermore, we observed that the *in vitro* stimulation of the TAS2R38 receptor with a specific agonist (PROP) modulates fundamental processes of adipocyte metabolism, such as delipidation and lipid accumulation. Interestingly, the PROP-induced decrease in lipid accumulation up-regulated TAS2R38 expression and at the same time, negatively affected the expression of genes involved in the differentiation process (*i.e.* FASN, PPARγ and GLUT4). This finding supports the hypothesis that TAS2R38, like perhaps other taste receptors here not investigated, can modulate adipocyte functions. The few data available in literature on taste receptors in adipose tissue, derive from studies conducted on mice and murine cell lines [18–21,28,29]. Previous studies demonstrated that during adipogenesis, adipocytes overexpress the sweet taste receptors and that these receptors mediate the inhibitory effect of artificial sweeteners on adipogenesis [20]. Furthermore, mice knockout for sweet taste receptors and fed on an “obesogenic” diet, accumulate less adipose tissue and composed by smaller adipocytes compared to wild type mice [28,29]. The signalling modulation of bitter taste receptor to the membrane cholesterol content [30] and the demonstrated interaction between fat (CD36) and bitter-taste receptors [31] represent additional evidence for a role of TAS2Rs in lipid stores modulation in adipose cells. Although the *in vivo* supplementation with quinine and caffeine is able to decrease the size of mouse adipocytes [32], the *in vitro* effects of quinine in mice are less clear. It was reported that quinine stimulates adipogenesis through a way partially mediated by the bitter taste receptor T2R106 [21], but also that the treatment of pre-adipocytes with quinine decreased differentiation into mature adipocytes [18]. Our results point to a lypolytic and anti-adipogenic effect of quinine; however, the significant cytoxic effect observed with the highest concentration of this substance may requires that the appropriate concentrations be identified for clinical use with a precise pharmacologic study. The effect of PROP in promoting the delipidation of differentiated adipocytes and the inhibition of lipid accumulation during adipocyte differentiation, suggests that TAS2R38 is involved in lipid mobilization. It is tempting to speculate that TAS2R38 may participate in counteracting the nutrients overload to which the adipose cells of obese subjects are frequently exposed.

The overexpression of TAS2R38 in adipocytes of obese subjects further supports the biological role of taste receptors in human adipose tissue. This overexpression may be a consequence of the adipocyte hypertrophy that occurs following the expansion of the adipose tissue or represents a new regulatory mechanism. In diet-induced obese mice, the oral administration of a bitter taste receptor ligand decreases weight, fat mass, inflammatory markers, increased energy expenditure, and improves glucose tolerance, insulin sensitivity and lipid profile [33]. These benefits were ascribed to the stimulation of GLP-1 release by entero-endocrine cells; however, we could now hypothesize that changes in adipocyte metabolism might have contributed to the beneficial effects of bitter taste agonists. The adipose tissue bitter taste receptors may also be involved in the benefits induced by the bitter herbal substances, used for centuries in the traditional Chinese medicine to treat metabolic, hearth and digestive diseases [34].

In conclusion, TAS2R38 bitter taste receptor are overexpressed in adipocytes of obese individuals and involved in adipocyte metabolism. This finding opens an exciting new field in the obesity research. Further research on hormone-sensitive lipase activity and free fatty acid/glucose uptake pathways regulation by bitter agonists will help to understand whether the stimulation of taste receptors may be a therapeutic strategy to control the lipid accumulation in adipocytes.
